# Novel use of syndromic surveillance to monitor the impact of synthetic cannabinoid control measures on morbidity

**DOI:** 10.1186/s40621-019-0210-2

**Published:** 2019-07-08

**Authors:** Michelle L. Nolan, Amy Ehntholt, Thomas Merrill, Don Weiss, Ramona Lall, Denise Paone

**Affiliations:** 1 Bureau of Alcohol and Drug Use Prevention, Care, and Treatment, New York City Department of Health and Mental Hygiene, 42-09 28th Street, 19th Floor, Queens, NY 11101 USA; 2 Department of Social and Behavioral Sciences, Harvard T.H. Chan School of Public Health, Boston, MA USA; 3 Office of General Council, New York City Department of Health and Mental Hygiene, Queens, NY USA; 4 Bureau of Communicable Diseases, New York City Department of Health and Mental Hygiene, Queens, NY USA

**Keywords:** Syndromic surveillance, Synthetic cannabinoid, Surveillance, Public health

## Abstract

**Background:**

Using data from syndromic surveillance, the New York City Department of Health and Mental Hygiene (DOHMH) identified an increase in the number of emergency department (ED) visits related to synthetic cannabinoids. Syndromic surveillance data were used to target community-level interventions and assess the real-time impact of control measures in reducing synthetic cannabinoid (“K2”)-related morbidity.

**Methods:**

From April 2015 through September 2015, DOHMH implemented 3 separate interventions to reduce K2-related morbidity by limiting the availability of K2 products. Difference-in-difference analyses compared pre- and post-intervention differences in cannabinoid-related ED visit rates between neighborhoods and controls for Interventions A and B. City-wide count data were used to compare K2-related ED visits before and after Intervention C.

**Results:**

Syndromic data showed a reduction in K2-related ED visits following the 3 interventions. Respective decreases in rates of synthetic cannabinoid-related ED visits of 33 and 38% were detected at the neighborhood-level due to Interventions A and B, respectively. A decrease of 29% was calculated at the city level following Intervention C.

**Conclusions:**

In addition to identifying emerging public health concerns, syndromic data can provide valuable real-time evidence on the effectiveness of public health interventions.

**Electronic supplementary material:**

The online version of this article (10.1186/s40621-019-0210-2) contains supplementary material, which is available to authorized users.

## Background

In July 2014, using near real-time emergency department (ED) data from its syndromic surveillance system, the New York City Department of Health and Mental Hygiene (DOHMH) identified an increase in the number of ED visits related to synthetic cannabinoids (or “K2”) (New York City Department of Health and Mental Hygiene 2014). K2 refers to a class of drugs that affect the same brain receptors as cannabis, but are made from chemicals not derived from the cannabis plant. Use of K2 has been associated with increased ED visits in New York City and nationally, with patients presenting with a variety of symptoms including lethargy, confusion, respiratory depression, and agitation (New York City Department of Health and Mental Hygiene 2018; Trecki et al. 2015). ED syndromic surveillance data are transferred daily from all New York City emergency departments to DOHMH and include information on the primary reason (chief complaint) for the ED visit, as well as patient age, gender, and ZIP code of residence (Nolan et al. 2017).

The increases identified in ED syndromic surveillance data suggested that K2-related ED morbidity was clustered in a few neighborhoods; in New York City, ZIP codes are nested within neighborhoods (New York City Department of Health and Mental Hygiene 2015). The public health investigation determined that K2 products were sold at a limited number of community grocery stores and smoke shops. This information informed a public health approach to reducing K2-related morbidity, which included the release of a health advisory, distribution of educational materials, and product removal (Nolan et al. 2016).

Although the New York State Department of Health passed an emergency regulation in 2012 making these products illegal, their possession was classified a low-level offense meaning police were required to witness a sale and know that the product sold contained synthetic cannabinoids in order to make an arrest. This requirement—and the limited penalties for an offense—made enforcing the regulation challenging.

As part of the strategy to reduce K2-related morbidity, ED syndromic surveillance data were used to identify neighborhoods for interventions. Neighborhoods that were disproportionately affected by K2-related morbidity, as indicated by ED syndromic surveillance data, were prioritized for control measures aimed at limiting product availability (Nolan et al. 2016).

Since enforcing the existing regulation was challenging, DOHMH issued a Commissioner’s Order on April 17, 2015 declaring K2 products to be a public health nuisance and ordering stores to cease and desist from selling K2 products at any time. Stores that failed to comply were in violation of the New York City Health Code—a misdemeanor subject to higher penalties. Enforcing the Commissioner’s Order required multiple steps and the cooperation of several city agencies.

Because many of the stores selling K2 products were licensed tobacco retailers, they were regulated by the New York City Sheriff’s Office and the Department of Consumer Affairs. Inspections were conducted simultaneously at multiple stores within the same neighborhood. Inspection teams consisted of representatives from the New York City Sheriff’s Office, the Civil Enforcement Unit, New York City Police Department, the Department of Consumer Affairs, and DOHMH (Nolan et al. 2016). Untaxed cigarettes were found at many of the stores, leading to broader inspections. Any identified K2 products found during these searches were removed, and the store was cited for violating the Commissioner’s Order that had been served prior to the inspection.

We describe the novel use—and the value—of ED syndromic surveillance data in measuring the real-time impact of interventions aimed at reducing the elevated rates of K2-related morbidity.

## Methods

The effects of three control measures (interventions) were assessed separately using ED syndromic surveillance data. Intervention A consisted of serving of Commissioner’s Orders to 34 stores in a single neighborhood; intervention B consisted of the removal of K2 products from 4 stores in a single neighborhood; and intervention C involved the removal of more than 200 kg of synthetic compounds and 150,000 packets of packaged K2 products coupled with the inspection of more than 80 stores across New York City.

ED visits related to K2 were identified using the free-text chief complaint field (Nolan et al. 2017; Additional file 1). Although discharge diagnosis codes (International Classification of Diseases, Tenth Revision, Clinical Modification [ICD-10-CM]) are included in the data, there are no ICD-10-CM codes specific to synthetic cannabinoids. Using the patient’s ZIP code of residence, we limited the analysis to residents of New York City and assigned each K2-related ED visit to either the intervention neighborhood or a control neighborhood. For Interventions A and B, the intervention neighborhood was defined as a single residential ZIP code in New York City. We used two different control groups: a neighborhood control group consisting of three ZIP codes with large numbers of ED visits prior to the intervention (but did not receive the intervention) and a city control group which included all residential ZIP codes in New York City that did not receive the intervention. Intervention C took place across New York City; therefore, all residential ZIP codes were defined as receiving the intervention.

K2 ED visit count data for the ten days preceding the intervention were compared with counts for the ten days following the intervention. Data from the date of the interventions were censored from the analyses. K2 ED visit rates per 100,000 residents were calculated by dividing the number of visits by the neighborhood’s population aged 15–84, using DOHMH intercensal estimates based on the 2010 US Census for 2017 (NYC DOHMH population estimates 2016). We calculated the difference in the outcome (rate of K2-related ED visits) in the intervention neighborhood before and after the intervention, minus the difference in the outcome in the control neighborhood before and after the intervention. Statistical testing for significance was not performed due to the limited number of data points.

## Results

Difference-in-difference (DID) revealed reductions in rates of ED visits following each of the interventions (Table 1: A, B, and C).

**Table 1:** Impact of synthetic cannabinoid control measures on emergency department (ED) visits, New York City, 2015

	ED visit 10 days pre-intervention		ED visits 10 days post-intervention		
	Number	Rate	Number	Rate	Difference
Intervention					
A (Treatment)	33	117.2	22	78.1	− 39.1
A (Neighborhood control)	30	23.6	29	22.8	−0.8
A (City control)	116	1.7	111	1.6	−0.1
*Difference-in-difference (Treatment - Neighborhood)*					−38.3
*Difference-in-difference (Treatment - City)*					−39.0
B (Treatment)	40	142.0	24	85.2	−56.8
B (Neighborhood control)	47	36.9	43	33.8	−3.1
B (City control)	341	5.0	327	4.8	−0.2
*Difference-in-difference (Treatment - Neighborhood)*					−53.7
*Difference-in-difference (Treatment - City)*					−56.6
C (Treatment)	330	4.8	232	3.4	−1.4

Notes. Rates expressed per 100,000 New York City residents

A-Commissioner’s Order served to stores in a single ZIP code

B-Product removal in a single ZIP code

C-Citywide product removal

Compared to the neighborhood control, the DID coefficients (− 38.3; − 53.7) suggested a 33%-decrease in rates of K2-related ED visits due to the serving of Commissioner’s Orders (Intervention A: − 38.3/117.2) and a 38% decrease due to the removal of products in 4 stores (Intervention B: − 53.7/142.0). Among both types of control groups, rates of K2-related ED visits were similar pre- and post-intervention Table 1). Analysis of ED syndromic surveillance data indicated a 29%-reduction in the rate of K2-related ED visits at the city-level immediately following widespread product removal from more than 80 stores across New York City (Intervention C: − 1.4/4.8).

## Discussion

We used ED syndromic surveillance data in a novel way to assess the impact of three control measures to reduce K2-related morbidity. Results from analyses of ED syndromic surveillance data suggested a decrease in rates of ED visits following serving stores with Commissioner’s Orders (Intervention A). Additional reductions in rates of K2-related ED visits were seen at the neighborhood-level following neighborhood-specific product removal efforts (Intervention B) and at the city-level following city-wide product removal efforts (Intervention C).

Although DOHMH monitors drug-related data on a daily basis to identify trends in drug-related morbidity, we had not previously used the data to evaluate the impact of an intervention to reduce drug-related morbidity. The real-time nature (most ED data are received hourly but all at least every 24 h) of syndromic data allows the effect of an intervention to be measured immediately.

Product removal efforts were labor-intensive and required the cooperation of multiple agencies. Identifying and quantifying the immediate impact of these interventions was immensely helpful in galvanizing future support for similar efforts.

## Public health implications

ED syndromic surveillance data represent a valuable, often underutilized, tool for measuring in real-time the effect of public health interventions.

## Additional file

Additional file 1: ED syndromic surveillance definitions for synthetic cannabinoids. (DOCX 12 kb)

## Abbreviations

DID: Difference-in-difference
DOHMH: Department of health and mental hygiene
ED: Emergency department
K2: Used as a synonym for synthetic cannabinoid
