# Supplementary material for: Novel use of syndromic surveillance to monitor the impact of synthetic cannabinoid control measures on morbidity
Source: Inj Epidemiol. 2019 Jul 8;6:33. doi: 10.1186/s40621-019-0210-2 (PMC6613244; doi:10.1186/s40621-019-0210-2)
Supplement: Supplementary file 1 — ED syndromic surveillance definitions for synthetic cannabinoids. (DOCX 12 kb) [file 40621_2019_210_MOESM1_ESM.docx]

/*---------------------------------------------------------------------------*\

| Supplementary material: |

| Novel use of syndromic surveillance to monitor the impact of |

| synthetic cannabinoid control measures on morbidity |

| |

| |

| Created by: Michelle L. Nolan, MPH |

\*---------------------------------------------------------------------------*/

/*THC*/

If prxmatch("/THC|MAR[AI]JUANA|MAR[AI]JANA|MARIJUANNA|MARJUANA|MAR[AI][HJ]ANA|

MARIJUAN|MARIJUNANA|MARIJAUANA|MARIJUANA|MARAJUNA|MARIJIUANA/", CC) >**0**

Then THC=**1**;

Else THC=**0**;

If THC=**0** and prxmatch("/MARJIUANA|MARAJANA|CANNABIAS|CANABIS|CANNIBIS|CANNIBIS|CANNIBUS|

CANABUS|CANIBIS|CANNIBAS|CANNAVIS|CANNBIS|CANNABUS|CANABISS|CANABES|WEED|POT|MJ/", CC)>**0**

Then THC=**1**;

If THC=**1** and prxmatch("/[A-Z]POT|POT[A-Z]|[A-Z]MJ|MJ[A-Z]|[A-Z]THC|THC[A-Z]|HOT POT|POT HOLE|WEED WACKER|BURN/", CC)>**0**

Then THC=**0**;

/*SYNTHETIC MARIJANA*/

If prxmatch("/SYNTHETIC|K2|K-2|K-TWO|K TWO|SPICE|BLACK MAMBA|LEGAL HIGH|ZOMBIE|VOODOO DOLL|BLACK MAGIC|CLOUD 10|CLOUD 9|INCENSE|POT POTPOURRI|WICKED X/", CC)>**0**

Then SYN_MJ=**1**;

Else SYN_MJ=**0**;

If THC=**1** and prxmatch("/LEGAL/", CC)>**0**

Then SYN_MJ=**1**;

If SYN_MJ >**0** and prxmatch("/HOSPICE|SPICY|SPICEY|WEEK|SPICES|K2[0-9]|[A-Z]K TWO|[A-Z]K-2|[A-Z]K TWO/", CC)>**0**

Then SYN_MJ=**0**;
